# Interleukin-17 producing cells in swine induced by microbiota during the early postnatal period - a brief research report

**DOI:** 10.3389/fimmu.2023.1214444

**Published:** 2023-09-20

**Authors:** Hana Stepanova, Marketa Scheirichova, Jan Matiasovic, Karolina Hlavova, Marek Sinkora, Katerina Stepanova, Martin Faldyna

**Affiliations:** ^1^ Department of Infectious Diseases and Preventive Medicine, Veterinary Research Institute, Brno, Czechia; ^2^ Laboratory of Gnotobiology, Institute of Microbiology of the Czech Academy of Sciences, Novy Hradek, Czechia

**Keywords:** interleukin-17, swine, postnatal, T cells, germ-free

## Abstract

Interleukin-17A (IL-17) is a pro-inflammatory cytokine involved in the immune response to many pathogens playing also a role in certain chronic and autoimmune diseases. The presented study focused on the early postnatal development of IL-17 producing cells in swine. In agreement with previous studies, αβ T-helper (CD3^+^CD4^+^) and γδ T (CD3^+^TCRγδ^+^) cells were found to be the major producers of IL-17. In newborn conventional piglets, αβ T-helper cells positive for IL-17 were almost undetectable, but their frequency increased markedly with age in all issues examined, i.e., blood, spleen, and mesenteric lymph nodes (MLN). Additional analyses of CD8 and CD27 expression showed that the main αβ T-helper producers of IL-17 has CD8^+^CD27^-^ phenotype in all tissues. IL-17 positive CD8^+^CD27^+^ αβ T-helper subpopulation was found only in blood and spleen. The production of IL17 in CD8^-^CD27^+^ αβ T-helper cells was always minor. In contrast, γδ T cells positive for IL-17 did not show a similar age-dependent increase in blood and spleen, whereas they increased in MLN. Because of the age-dependent increase in conventional animals, we included a comparison with germ-free piglets to show that the increase in IL-17 positive cells was clearly depended on the presence of the microbiota as the production in germ-free animals was negligible without any age-dependent increase.

## 1 Introduction

Interleukin-17A (hereafter IL-17) is a pro-inflammatory cytokine involved in the immune response to many pathogens and appears to play a role in certain chronic and autoimmune diseases. It is a 17 kDa protein and belongs to the cytokine family, which includes six molecules named IL-17A to IL-17F with different functions. The receptor for IL-17 has been found in almost all tissues (1). The discovery of IL-17 modified the Th1/Th2 paradigm and led to the emergence of a new Th effector subgroup called Th17 (2, 3). Subsequently, other cell types have been reported to produce IL-17, including T cells with γδ T cell receptor (TCR γδ) (4), NK cells, and lymphoid tissue inducer-like cells (LTi-like cells) (5, 6). Furthermore, CD8^+^ T cytotoxic cells and NKT cells have been identified as potential sources of IL-17 (7–9).

Most data on IL-17 producing cells have been obtained in mice and humans. However, the last two decades have produced quite a number of reports in pigs. Recombinant porcine IL-17 showed similar biological effects as IL-17 from humans and mice (10), and the ability of the porcine immune system to polarize toward the Th17 axis has been documented (11). IL-17 mRNA has been detected in porcine tissues, including blood, heart, skin, lymph nodes (10), and lung (12). The finding of cross-reactive antibody enabled the phenotyping of IL-17 producing cells, which include CD3^+^CD4^+^ and CD3^+^TCRγδ^+^ (13). Recently new anti-IL-17 mAbs expanded opportunities for the cytokine research in swine (14). IL-17 producing Th cells have already been identified in other tissues, including lung and lymph nodes (15). Finally, γδ T splenocytes have also been found to be a source of IL-17 (16).

The presence of IL-17 in swine has also been reported in the context of some infection models. The involvement of the cytokine has been observed during viral infections, including African swine fever (17) and influenza (18). The cytokine is involved in antibacterial immunity, for example during infection with enterotoxigenic *Escherichia coli* (19), mycoplasma (20), or infection with *Actinobacillus pleuropneumoniae* (15). Also *Glaesserella parasuis* infections upregulated IL-17 production both *in vitro* and *in vivo* and IL-17 expression positively correlated with degree of pathological tissue injury (21). It has also been reported as a mediator during scabies infection (22, 23). IL-17 has also been observed during the immune response after vaccination (24), and an increase in IL-17 has also been associated with ultra-early weaning (25).

On the other hand, there is a lack of information on IL-17 mediated immunity in the context of early postnatal ontogenesis, which is the aim of this work. Because there is a significant increase in IL-17 producing cells within the first weeks of life, we tested the hypothesis whether the spontaneous colonization with microbiota (commensal and environmental microorganisms) plays an essential role in the induction of IL-17 producing cells.

## 2 Materials and methods

### 2.1 Animals

Conventional (CV) and germ-free (GF) piglets were used for the study. All animals were from herds with a good epidemiological situation. Large White sows were a source of CV piglets. The CV experiments were performed in two independent runs at the Veterinary Research Institute (Brno, Czech Republic). Piglets were nursed throughout the course of the experiment and were euthanized on days of life 0, 7, 14, and 28 by bleeding the brachial artery under deep anesthesia (tiletamine 2mg + zolazepam 2 mg + ketamine 2 mg + xylazine 2 mg/per kg of body weight). Samples from 6-month-old pigs were collected from animals exsanguinated after electrical stunning at the institutional experimental slaughterhouse. Animal treatment and use protocols were approved by the Ethics Committee of the Veterinary Research Institute in accordance with the guidelines of the Animal Protection Act and were subsequently approved by the Branch Commission for Animal Welfare of the Ministry of Agriculture of the Czech Republic.

A source of GF piglets were Minnesota miniature/Vietnamese–Asian–Malaysian crossbred piglets held at the Laboratory of Gnotobiology (Novy Hradek, Czech Republic). Piglets were recovered from gilts by sterile hysterectomy on day 112 of gestation as previously described (26–29). After recovery, GF piglets were kept in sterile isolator units under GF conditions and were hand-fed by γ-irradiated sterile 6%-fat cow milk reconstituted from evaporated 9%-fat concentrate by dilution with water (30). Piglets were euthanized by intracardiac puncture under general anesthesia on days 7 and 28 after the day of recovery. All these experiments were approved by the Ethics Committee of the Institute of Microbiology of the Czech Academy of Science according to the guidelines of the Animal Protection Act.

### 2.2 Sample collection, cell isolation and stimulation

Blood was collected from the jugular vein and immediately heparinized using 25 IU/mL of sodium heparin (Zentiva). The spleen and mesenteric lymph nodes (MLN) were collected immediately after euthanasia. A single-cell suspensions were obtained from the tissues using a 100 µm cell strainer and then washed three times with phosphate buffered saline (PBS). Cells were used for stimulation at a concentration of 10^6^/ml in RPMI-1640 medium (Sigma–Aldrich) supplemented with antibiotics (100 IU/ml penicillin, 100 μg/ml streptomycin) and 10% fetal bovine serum (FBS). Blood was diluted 1:1 with RPMI-1640 medium supplemented with antibiotics. Samples were stimulated with 15 nM/ml phorbol-myristate-acetate (PMA; Sigma–Aldrich) and 1 μg/ml ionomycin (Sigma–Aldrich) for 5 hrs. For flow cytometry, samples were stimulated in the presence of a protein transport inhibitor, brefeldin A (10 μg/ml, Sigma–Aldrich). Unstimulated control samples were cultivated with brefeldin A only. After ammonium chloride-mediated lysis of erythrocytes and washing steps with PBS, samples were stained for flow cytometry or stored in Tri-Reagent RT (Molecular Research Center) for subsequent preparation for quantitative reverse transcription PCR (RT-qPCR).

### 2.3 Sample staining, flow cytometry and cell sorting

Flow cytometry analysis was performed using a combination of IL-17 intracellular staining and surface markers. The primary mouse anti-pig antibodies were as follows: CD3ϵ (PerCP-Cy™5.5, BB23-8E6-8C8, BD Biosciences), CD2 (unconjugated, PG168A, IgG3, WSU), TCRγδ (unconjugated, PGBL22A, IgG1, WSU), CD4 (unconjugated, 10.2H2, IgG2b, WSU), CD8α (unconjugated, 76-2-11, IgG2a, WSU). As secondary antibodies, isotype-specific, fluorochrome-labelled goat anti-mouse antibodies were used: Alexa Fluor 488 (IgG2a, Invitrogen-Termo Fisher Scientific), Alexa Fluor 647 (IgG2b, Invitrogen-Termo Fisher Scientific), Dylight 405 (IgG1, BioLegend), Phycoerythrin (IgG1, Invitrogen-Termo Fisher Scientific). Samples were stained as previously described (13). Briefly, surface markers were stained with unconjugated primary and then fluorochrome-conjugated secondary antibodies. After a blocking step with 10% mouse serum, PerCP-Cy5.5-conjugated anti-CD3 antibody was added. Prior to the fixation step, the LIVE/DEAD™ Fixable Yellow Dead Cell Stain Kit viability probe (for 405 nm excitation) was added. Samples were then fixed and permeabilized using an Intrastain kit (Dako). During the permeabilization step, anti-human cross-reactive PE-conjugated anti-IL-17 antibody (PE, SCPL1362, BD Biosciences) or isotype control antibody (mouse IgG1 negative control PE-conjugated; BD Biosciences) was added. Flow cytometry was performed using an LSR Fortessa flow cytometer, operated by Diva software, version 6.0 (BD Biosciences). Doublets (defined by plotting the width against the area of forward scatter) and dead cells were excluded from the analysis.

Only samples stained with surface markers were used for fluorescence-activated cell sorting (FACS). Primary and secondary antibodies described above and mouse anti- pig CD27 (unconjugated, B30C7, IgG1, BioRad) were used. Dead cells and doublets were excluded from the sorting. Samples were sorted on the BD FACS Aria Fusion operated by Diva software, version 8.0 (BD Biosciences) under the following conditions: nozzle size 70µm, pressure 70 psi, frequency 87 kHz, four-way purity sort mask. The sorted cells were collected into 5 ml tubes containing RPMI-1640 medium supplemented with antibiotics and 10% FBS. After sorting, the tubes were centrifuged and the cells were resuspended in fresh medium for subsequent stimulation with PMA and ionomycin. After five hours of stimulation, cells were centrifuged and the pellet was lysed with Tri-reagent RT for further IL17mRNA analysis by qRT-PCR.

### 2.4 Quantitative reverse transcription PCR

The isolation of RNA, reverse transcription, and RT-qPCR were performed according to previously described methods (31). Briefly, the RNA phase was isolated from Tri-Reagent RT homogenate mixed with bromoanisole with subsequent RNA purification using an RNeasy Mini Kit (Qiagen). RNA was then reverse transcribed with M-MLV reverse transcriptase (Invitrogen- Termo Fisher Scientific) using oligo-dT primers. Measurements were performed using the QuantiTect SYBR Green PCR Kit (Qiagen), LightCycler 480 II with 384-well plate block (Roche) and Innovadyne Nanodrop robot (IDEX Health & Science LLC). Hypoxanthine phosphoribosyltransferase 1 (HPRT1) was used as a housekeeping reference gene. The threshold cycle values (Ct) of the gene of interest (IL-17) was first normalized to the Ct value of HPRT reference mRNA (ΔCt) and then the normalized mRNA levels were calculated as [1/(2Ct target gene)]/[1/(2Ct HPRT)]. The normalized mRNA levels of IL-17 mRNA are expressed as “HPRT units”. Primers used in the study were adopted from Stepanova et al., 2012 (13).

### 2.5 Statistical analysis

Data were analysed using nonparametric ANOVA (Kruskal–Wallis test). Differences between CV and GF piglets were analysed using the nonparametric Mann-Whitney test. For both, p-values < 0.05 were considered to indicate significant differences. Statistical analysis was performed using GraphPad Prism software (GraphPad Software, version 5.04).

## 3 Results

### 3.1 Postnatal changes in IL-17 expression and development of IL-17 producing cells

**Figure 1:**
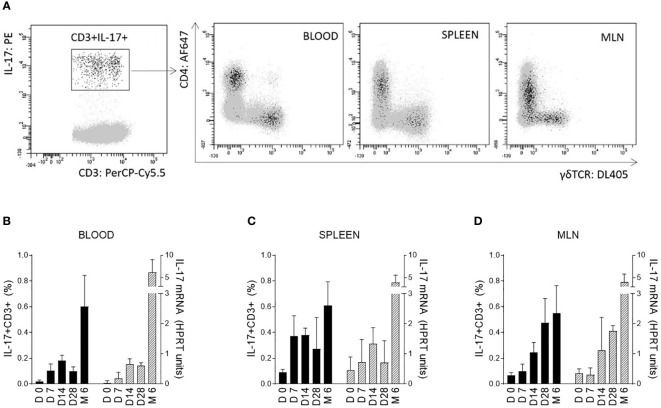
IL-17 producing T cells and correlation with age of pigs. IL-17 producing T cells (CD3^+^) are shown on a representative image **(A)**. Cells isolated from blood, spleen, and MLN of 6-month-old CV pigs were stimulated with PMA and ionomycin, and CD3^+^ live singlet cells were analysed for expression of IL-17 on CD4^+^ and TCRγδ^+^ cells **(A)**. The level of IL-17 during postnatal development was also evaluated **(B–D)**. Cells were isolated from the blood **(B)**, spleen **(C)** and MLN **(D)** of CV pigs from four age categories: day (D0, D7, D14, D28) and 6 months (M6) postpartum. Left Y axes represent percentages of IL17^+^CD3^+^ cells (black bars), whereas right Y axes represent levels of IL-17 transcripts as evaluated by RT-qPCR relative to IL-17mRNA HPRT units (grey bars). Data are expressed as mean (columns) and SEM (error bars), n=6.

Changes in IL-17 levels during the early postnatal period were evaluated for blood cells and selected tissues immediately after birth (day 0) and 7, 14, and 28 days after birth. These data were compared with those from 6-month-old pigs. The ability of cells to produce IL-17 after polyclonal stimulation was measured at the mRNA level using RT-qPCR and by flow cytometry as the percentage of IL-17 positive cells. Differentiation was based on the five surface markers CD3/CD2/CD4/CD8/TCRγδ in combination with intracellular IL-17 staining after stimulation with PMA and ionomycin. The αβ T-helper (CD3^+^CD4^+^) and γδ T (CD3^+^TCRγδ^+^) cells were found to be the strongest IL-17 producers (**Figure 1A**). The total percentage of IL-17 positive cells was evaluated within all T cells (defined as CD3+) according to the gating strategy shown in **supplementary data** (**Figure S1** – gating strategy).

The significant increase in IL-17 was confirmed at both mRNA and protein levels during the early postnatal period for cells isolated from blood (**Figure 1B**), spleen (**Figure 1C**) and MLN (**Figure 1D**). The trend was similar in both blood and solid immune organs. The age dependent changes were evaluated by Kruskal-Wallis test (used to determine whether 5 different age groups are significantly different from each other on the variable “HPRT units of IL-17 mRNA” and “percentage of IL17+CD3+ cells”) with following results for HPRT units of IL-17 mRNA (blood: p < 0.0001, spleen: p = 0.0014 and MLN: p < 0.0001) and for percentage of IL17^+^CD3^+^ cells (blood: p < 0.0001, spleen: p = 0.0014 and MLN: p < 0.0001). An increasing trend apparently continued during aging with a substantial augmentation between 28-day-old and 6-month-old pigs. Relatively high individual variability was observed, especially in older animals.

### 3.2 Postnatal development of IL-17 producing αβ T-helper and γδ T cells

**Figure 2:**
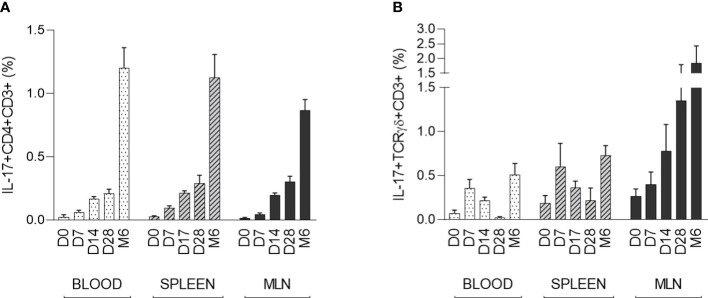
The percentage of IL-17 positive αβ T-helper and γδ T cells during the early postnatal period. The percentages of IL-17 positive αβ T-helper **(A)** and γδ T **(B)** cells are shown. Cells were isolated from the blood, spleen and MLN of CV pigs from four age groups: day (D0, D7, D14, D28) and 6 months (M6) postpartum. Samples were stimulated with PMA and ionomycin, and CD3^+^CD4^+^ or CD3^+^TCRγδ^+^ live singlet cells were analysed for expression of IL-17. The data are expressed as mean (columns) and SEM (error bars), n=6.

The strongest IL-17 producers αβ T-helper (CD3^+^CD4^+^) and γδ T (CD3^+^TCRγδ^+^) cells were evaluated in the context of early postnatal ontogenesis. The percentage of IL-17 producing cells was measured by flow cytometry according to the gating strategy shown in **Supplementary Data** (**Figure S1**). The data obtained demonstrated a significant increase in IL-17 producing αβ T-helper cells in blood, spleen, and MLN (**Figure 2A**). Statistical significance was found in all compartments analysed (Kruskal-Wallis, p < 0.0001). These IL-17 positive αβ T-helper cells were almost undetectable in newborn CV piglets. The first evidence of these cells occurred on day 7. The obtained data showed relatively low individual variability during the early postnatal period. The increase in IL-17 positive cells was gradual until day 28, followed by a substantial increase detectable in the group of 6-month-old pigs. In contrast to αβ T-helper cells, IL-17 positive γδ T cells were detectable in newborn piglets and exhibited different kinetics during early postnatal development (**Figure 2B**). A gradually increasing trend was found only in MLN (Kruskal-Wallis, p = 0.0129). The Kruskal-Wallis test also revealed significant differences in blood (p = 0.0033) and spleen (p = 0.0415). However, the percentage of IL-17 positive γδ T cells varied within age categories. Relatively high levels were found in spleen and blood at day 7, but low frequencies were detectable in these compartments at day 28, followed by an increase in 6 months-old pigs. The data on IL-17 positive γδ T cells were characterized by high individual variability. In addition, the phenotype of IL-17 producing γδ T cells was further analysed based on the expression of CD2, and only CD3^+^TCRγδ^+^CD2^-^ subpopulation was found as IL-17 positive (**Supplementary Data**, **Figure S2**).

**Figure 3:**
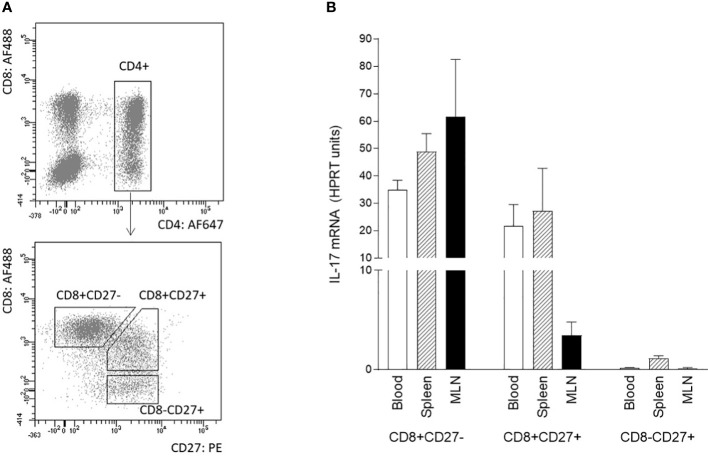
Phenotype of IL-17 producing αβ T-helper cells based on CD8 and CD27 expression. The phenotype of IL-17 producing αβ T-helper cells based on CD8 and CD27 expression was evaluated using sorted cells isolated from blood, spleen, and MLN of 6-month-old pigs. Three subpopulations of CD3^+^CD4^+^ cells were sorted: CD27^+^CD8^-^, CD27^+^CD8^+^ and CD27^-^CD8^+^ **(A)**. Sorted cells were stimulated with PMA and ionomycin and levels of IL-17 transcripts as evaluated by RT-qPCR relative to IL-17mRNA HPRT units **(B)**. Data are expressed as mean (columns) and SEM (error bars), n=4.

The differentiation/activation status of IL-17 producing αβ T-helper cells was also assessed by CD8α and/or CD27 expression. The combination of sorting followed by RT-qPCR was used because flow cytometry data did not provide entirely clear results about the IL-17 positivity of particular subpopulation. The following subpopulations of CD3^+^CD4^+^ cells were sorted according to the gating strategy shown in **Figure 3A**: CD27^+^CD8^-^, CD27^+^CD8^+^, and CD27^-^CD8^+^. Sorted subpopulations were then stimulated with PMA and ionomycin, and IL-17 transcripts were measured using RT-qPCR. The data obtained clearly showed that specifically the CD8 positive subpopulation was a source of IL-17 in all monitored compartments (**Figure 3B**). The IL-17 transcripts were almost undetectable in CD4^+^CD8^-^CD27^+^ subpopulation. Populations with both CD4^+^CD8^+^CD27^-^ and CD4^+^CD8^+^CD27^+^ phenotypes were proved as IL-17 sources in blood and spleen. In MLN, CD4^+^CD8^+^CD27^-^ cells exhibited higher IL-17 mRNA levels compared with their CD4^+^CD8^+^CD27^+^ counterparts.

### 3.3 Comparison of IL-17 producing T cells in GF and CV piglets

**Figure 4:**
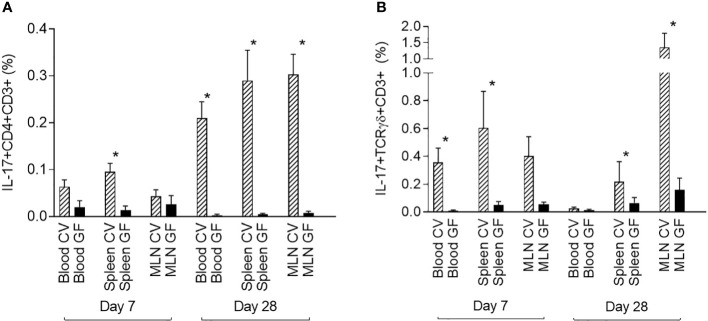
Comparison of IL-17 producing αβ T-helper and γδ T cells in GF and CV piglets. The percentage of IL-17 positive αβ T-helper **(A)** and γδ T **(B)** cells is shown. Cells were isolated from the blood, spleen, and MLN of CV and GF pigs from two different age groups (day 7 and day 28) after birth. Cells were stimulated with PMA and ionomycin, and CD3^+^CD4^+^ **(A)** or CD3^+^TCRγδ^+^ **(B)** live singlet cells were analysed for expression of IL-17. Data are expressed as mean (columns) and SEM (error bars). Data were analysed using Mann-Whitney test, statistically significant differences between CV and GF group are marked in graphs (* p < 0.05).

Data from CV pigs were compared with data from GF piglets to demonstrate the role of the microbiota in the induction of IL-17 producing T cells. One-week-old (day 7) and one-month-old (day 28) piglets were included in this part of the study. The GF conditions had a significant impact on the induction of IL-17. The data showed a clear impact of microbiota on the postnatal development of both IL-17 positive αβ T-helper (CD3+CD4+) and γδ T (CD3+TCRγδ+) cells (**Figure 4**). In contrast to the data from CV piglets, IL-17 positive αβ T-helper cells were almost undetectable at both time points, day 7 and day 28 postpartum (**Figure 4A**). This trend was evident in all compartments examined. The similar impact was observed for γδ T cells (**Figure 4B**). Significant differences between CV and GF piglets evaluated by Mann-Whitney test were found in blood and spleen at day 7, and in spleen and MLN at day 28.

## 4 Discussion

IL-17 is a pro-inflammatory mediator of both antigen-specific and nonspecific innate responses. In the study, we present an overview of the ability of piglet immune cells to produce IL-17 in the early postnatal period. Cytokine levels increased significantly during the first month of life in both blood and solid immune organs (spleen and MLN). This trend indicates that an immune response associated with IL-17 is induced as a consequence of contact with microorganisms, which we have partially proved using a GF piglet model. This is fully consistent with the recent view on the role of IL-17 in αβ T cells (32). However, pigs belong to the γδ-high species with a much higher representation of γδ T cells than mice or humans (27, 28, 33). Comparison of the two major IL-17 producers in pigs suggests different aspects of αβ T-helper and γδ T cells development consistent with the overal review (34).

Differentiation of naive CD4^+^ cells into effector subsets (including Th17) occurs in peripheral tissues after previous positive and negative selection in the thymus. The peripheral stimulation is driven by the recognition of antigens presented by antigen-presenting cells on MHC molecules and by the cytokine environment, leading to the development of differentiated memory-type Th cells. In general, the number of memory Th17 lymphocytes is expected to increase with age. Our current data clearly showed that the proportion of IL-17 producing αβ T-helper cells increases significantly during early postnatal period.

Using GF piglets, we found that IL-17 producing αβ T-helper cells are undetectable in one-month-old GF pigs, while relatively high levels of these cells were detectable in CV piglets of the same age. The development of Th17 cells in relation to the microbiota was studied in detail in a mouse model. The microbiota driving Th17 cell differentiation has been clearly demonstrated by two research groups (35, 36). In our study, we confirmed the role of microbiota in the development of porcine Th17 cell, which is in agreement with the finding from the mouse model. Previously published data suggest that the development of activated and memory Th cells occurs as a result of a microbial stimulus in porcine tonsils (37). Similar results have been published on porcine intestinal T cells, where the proportion of CD4^+^CD8^+^ Th cells was significantly higher in CV piglets compared to GF animals (30). When porcine αβ T-helper cells were sorted based on CD8 expression in the study, their ability to produce IL-17 was proved only within the CD8^+^ subpopulation, which has been shown to be a memory/effector Th subset (29, 38). Based on data published about co-expression of CD8 and CD27, the following differentiation stages of porcine Th cells were defined: “naive cells” defined as CD8^-^CD27^+^, “effector memory cells” CD8^+^CD27^-^ and “central memory cells” CD8^+^CD27^+^ (39). Both memory phenotypes were detected in the study as sources of IL-17, but with different distributions in the analysed compartments. While both memory forms were detectable in blood and spleen, CD8^+^CD27^-^ effector memory cells predominated in MLN. Putative memory Th17^+^ cells (CD4^+^CD8α^dim^IL-17^+^) have been previously identified in lung and blood during the chronic phase of *Actinobacillus pleuropneumoniae* infections (15).

The development of γδ T cells (the second most potent population producing IL-17) differs significantly from that of αβ T-helper cells (40–42). Our current data shows that detectable numbers of IL-17 producing γδ T cells are already present in newborn piglets. This observation is supported by data from a study in mice in which IL-17 producing γδ T cells were detected in thymus during prenatal development (43). Finally, IL-17 production by TCRγδ^+^ cells has been shown to be induced by cytokines, predominantly IL-23 and IL-1 (44, 45).

In the study, a marked increase in IL-17 positive γδ T cells was observed during the first week in CV piglets, but their frequency did not show further increase as in IL-17 producing αβ T-helper cells. However, there is no increase in GF animals, so the amount of γδ T cells producing IL-17 also depends on the presence of microbiota. The microbial-dependence is in agreement with studies in other species (46, 47). Earlier findings indicate that although γδ T cells expand after infection, they also migrate to effector destination so that their numbers subsequently decrease to basal levels (48). In swine, three basic γδ T cell subpopulations have been identified based on CD8 and CD2 expression: CD2^+^CD8^+^, CD2^+^CD8^-^ and CD2^-^CD8^-^ (38, 41, 42). Their developmental and functional characterization have shown that CD2^−^ γδ T cells appear to represent a lineage-specific subset (27, 28). The production of IL-17 is associated with this CD2^-^ γδ T cell subpopulation, as also reported by Sedlak et al. (16). Nevertheless, these authors describe that IL-17 producing CD2- γδ T cells also express the CD8 marker, which contradicts the above findings of defining only 3 subpopulations based on CD8 and CD2 expression. It is important to note that peripheral γδ T cells express CD8αα homodimers, which are detected in low expression compared with the high CD8αβ expression on TCRαβ^+^ T cells (42). In such case, all CD8^lo^ γδ T cells are also CD2^+^. However, high-sensitivity photomultipliers may increase the dispersion of the CD8^-^ population into CD2^-^CD8^-^ and CD2^-^CD8^lo^ γδ T cells. This may be the reason why some results indicate that IL-17 producing γδ T cells have the CD2-CD8lo phenotype, whereas CD2+ γδ T cells are CD2+CD8hi and αβ T cells are then CD2+CD8bri. This is an issue that remains to be resolved, and the existence of a CD2-CD8lo population needs to be clearly proven or disproven. We therefore plan future experiments that would provide data to elucidate the discrepancies regarding CD8 subpopulations of porcine γδ T cells.

In conclusion, the proportion of IL-17 producing cells increases in pigs during the first month after birth and this process depends on contact with the microbiota. Two major IL-17 producing populations could represent two branches of immunity. The adaptive part represented by αβ T-helper cells, which gradually increase during life and exhibit a memory phenotype. The innate IL-17 producers are mainly formed by γδ T cells, which do not show such a clear age dependence, but are also clearly induced by the presence of microbiota.

## Data availability statement

The raw data supporting the conclusions of this article will be made available by the authors, without undue reservation.

## Ethics statement

The animal study was approved by Ethics Committee of the Veterinary Research Institute subsequently approved by the Branch Commission for Animal Welfare of the Ministry of Agriculture of the Czech Republic and by the Ethics Committee of the Institute of Microbiology of the Czech Academy of Science according to the guidelines of the Animal Protection Act. The study was conducted in accordance with the local legislation and institutional requirements.

## Author contributions

MF and HS contributed to conception and design of the study. JM, MSi and KS organized the animal part of the study, HS, KH and MSc organized laboratory part of the study. HS performed the statistical analysis. HS wrote the first draft of the manuscript. MF and MSi wrote sections of the manuscript. All authors contributed to the article and approved the submitted version.

## Supplementary material

Supplementary Figure 1 Gating strategy. IL-17 producing T cells (CD3^+^) are shown on a representative image. Cells isolated from blood of 6-month-old CV pigs were stimulated with PMA and ionomycin. Single cells were defined by plotting the width against the area of forward scatter and subsequently LIVE/DEAD negative cells were evaluated as live cells. T cells (CD3^+^) were defined from single live cells and analysed for expression of IL-17 on CD4^+^ and TCRγδ^+^ lymphocytes.

Supplementary Figure 2 The phenotype of IL-17 producing γδ T cells based on CD2 expression is shown on a representative image. Cells isolated from blood of 6-month-old CV pigs were stimulated with PMA and ionomycin. γδ T cells were gated according to gating strategy shown in **Supplementary Figure 1**. IL-17 expression is shown for TCRγδ^+^CD2^-^ and TCRγδ^+^CD2^+^ cells.
