# Supplementary figures and images for: Interleukin-17 producing cells in swine induced by microbiota during the early postnatal period - a brief research report
Source: Front Immunol. 2023 Sep 20;14:1214444. doi: 10.3389/fimmu.2023.1214444 (PMC10548118; doi:10.3389/fimmu.2023.1214444)

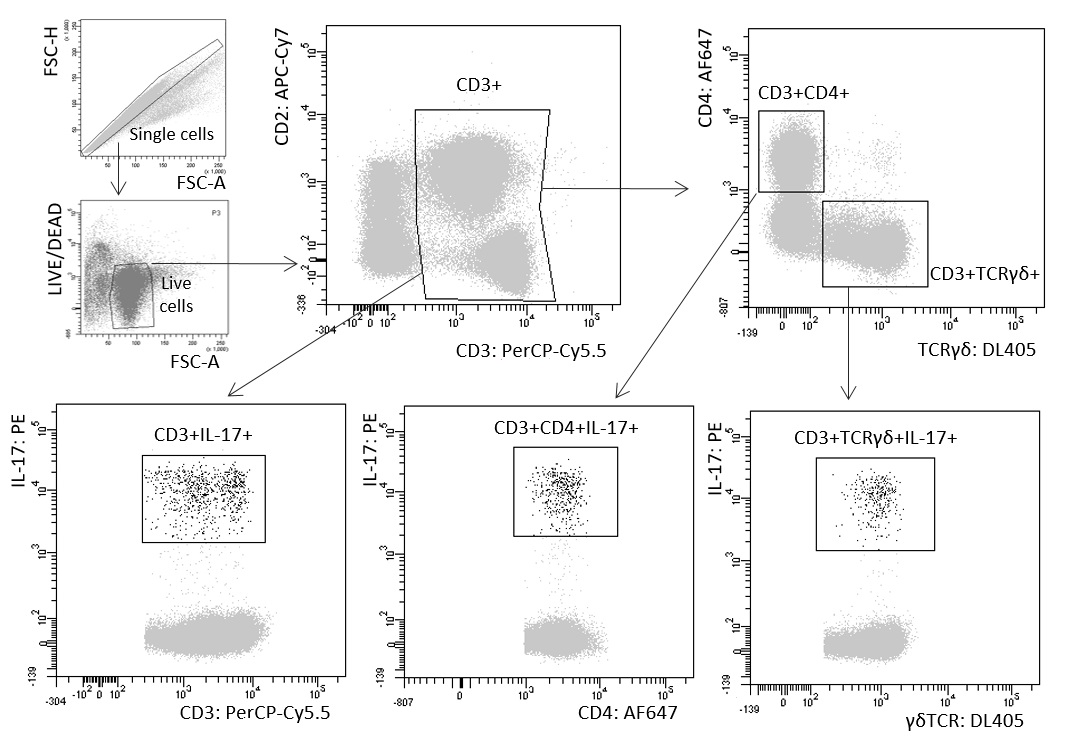

Supplement: Supplementary Figure 1 — Gating strategy. IL-17 producing T cells (CD3+) are shown on a representative image. Cells isolated from blood of 6-month-old CV pigs were stimulated with PMA and ionomycin. Single cells were defined by plotting the width against the area of forward scatter and subsequently LIVE/DEAD negative cells were evaluated as live cells. T cells (CD3+) were defined from single live cells and analysed for expression of IL-17 on CD4+ and TCRγδ+ lymphocytes. [file Image_1.jpeg]

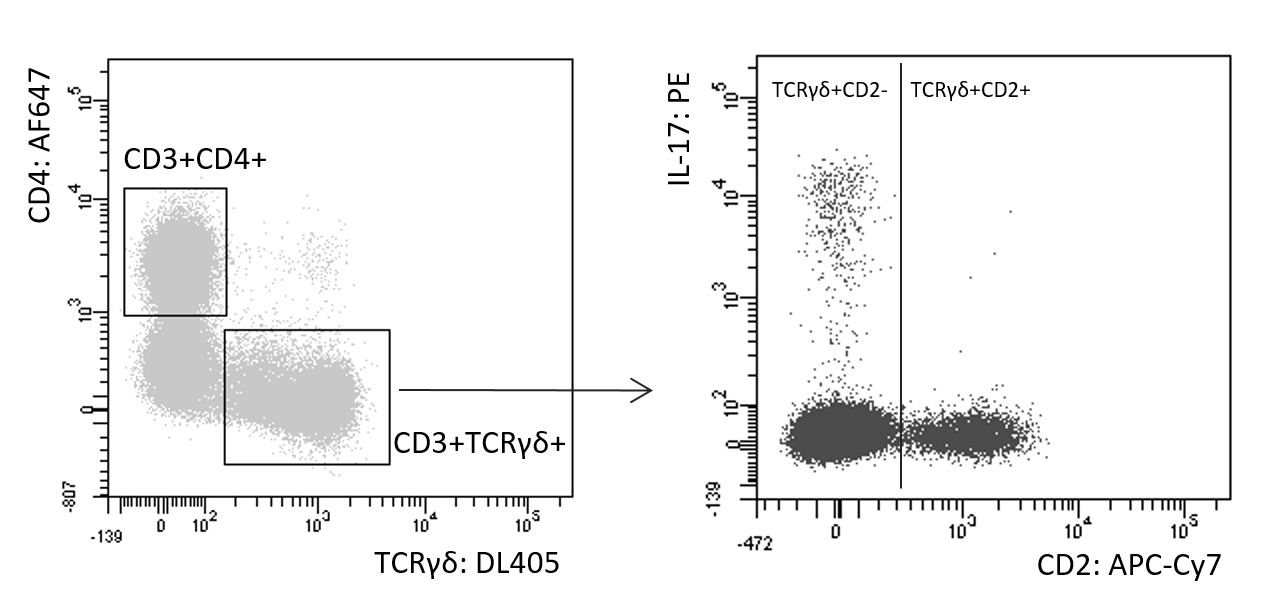

Supplement: Supplementary Figure 2 — The phenotype of IL-17 producing γδ T cells based on CD2 expression is shown on a representative image. Cells isolated from blood of 6-month-old CV pigs were stimulated with PMA and ionomycin. γδ T cells were gated according to gating strategy shown in Supplementary Figure 1 . IL-17 expression is shown for TCRγδ+CD2- and TCRγδ+CD2+ cells. [file Image_2.png]
